# Improved cell growth on additively manufactured Ti64 substrates with varying porosity and nanofibrous coating

**DOI:** 10.1016/j.heliyon.2024.e25576

**Published:** 2024-02-05

**Authors:** Ewin Tanzli, Tomasz Kozior, Jiri Hajnys, Jakub Mesicek, Bennet Brockhagen, Timo Grothe, Andrea Ehrmann

**Affiliations:** a Faculty of Engineering and Mathematics, Bielefeld University of Applied Sciences and Arts, 33619, Bielefeld, Germany; b Faculty of Mechatronics and Mechanical Engineering, Kielce University of Technology, 25-314, Kielce, Poland; c Department of Machining, Assembly and Engineering Metrology, Faculty of Mechanical Engineering, VSB – Technical University of Ostrava, 708 00, Ostrava Poruba, Czech Republic

**Keywords:** 3T3 cell line, Additive manufacturing, Laser metal fusion, Powder bed fusion, Cell culture, Nanofibers

## Abstract

3T3 Swiss albino mouse cells are often used in biotechnological applications. These cells can grow adherently on suitable surfaces. In our study, they were grown on different titanium substrates, comparing commercially available titanium sheets of grade 1 and grade 2, respectively, with Ti64 which was 3D printed with different porosity in order to identify potential substitutes for common well-plates, which could – in case of 3D printed substrates – be produced in various shapes and dimensions and thus broaden the range of substrates for cell growth in biotechnology and tissue engineering. In addition, thin layers of poly(acrylonitrile) (PAN) nanofibers were electrospun on these substrates to add a nanostructure. The common titanium sheets showed lower cell cover factors than common well plates, which could not be improved by the thin nanofibrous coating. However, the Ti sheets with nanofiber mat coatings showed higher cell adhesion and proliferation than pure PAN nanofiber mats. The 3D printed Ti64 substrates prepared by laser metal fusion, on the other hand, enabled significantly higher proliferation of (66 ± 8)% cover factor after three days of cell growth than well plates which are usually applied as the gold standard for cell cultivation ((48 ± 11)% cover factor under identical conditions). Especially the Ti64 samples with higher porosity showed high cell adhesion and proliferation. Our study suggests investigating such porous Ti64 samples further as a potential future optimum for cell adhesion and proliferation.

## Graphical abstract

**Figure:**
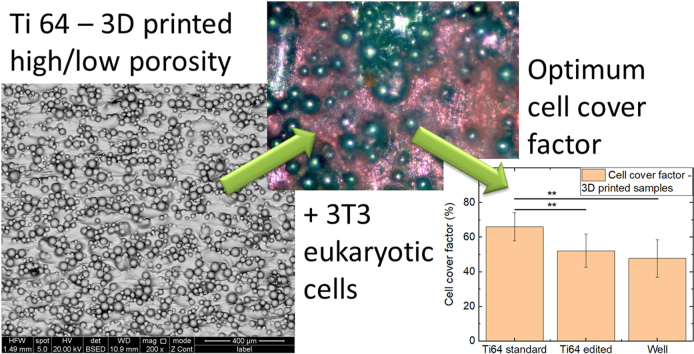

## 1 Introduction

Eukaryotic cell cultures are used in biotechnological processes for diverse applications such as tissue engineering [[1], [2], [3]], protein or antibody production [[4], [5], [6]], cytotoxicity examinations [7,8], and similar biotechnological and biomedical investigations [9].

Several cells can be grown in suspension; however, diverse adherent cell lines are also of high interest in biotechnology and biomedicine. Typically, adherent cells are grown in cell culture trays, roller bottles, or well-plates usually made of plasma-treated polystyrene. These tissue culture devices work well, but have relatively high production costs [10]. Cell adhesion can be promoted, e.g., by proteins added to the serum [11] or coating the cell culture flask with collagen [12]. For the growth of adherent cells in bioreactors, micro-carriers made out of modified dextran, cellulose or gelatin, or hollow fibers consisting of several synthetic polymers can be used as an alternative [13,14].

Generally, high-density adherent cell cultures are favorable for typical biotechnological applications [15]. Approaches to reach high cell densities include, e.g., packed-bed bioreactors or rolled scaffolds [[15], [16], [17]].

Recently, various new substrates have been suggested for the potential use in eukaryotic cell cultivation, mostly in order to improve cell adhesion and proliferation. Additive manufacturing belongs to the techniques which could easily form 3D shapes. 3D printing by fused deposition modeling (FDM) has been tested by different research groups for the potential use in cell cultivation. Lücking et al. investigated 3D printing materials with respect to their *in-vitro* biocompatibility by printing polyamide well-plates with different baffle geometries [3]. Sölmann et al. tested FDM printed substrates with different surface structures from poly(lactic acid) (PLA) and polyethylene terephthalate glycol (PETG) and found that Chinese hamster ovary (CHO) cells grew on all tested polymers [18]. Li et al. reported the growth of a MC3T3-E1 cell culture on 3D-printed PLA scaffolds with high porosity [19]. Especially for the application in bone tissue engineering, Melcová et al. used FDM printing to prepare samples from plasticized poly(3-hydroxybutyrate)/poly(d,l-lactide) 70/30 blend modified with tricalcium phosphate bioceramics and found non-cytotoxicity and biocompatibility [20]. Using stereolithography (SLA), Kreβ et al. investigated the cytotoxicity of different photopolymers on mesenchymal stem cells (MSCs) and suggested coating the samples with Parylene to protect the MSCs from toxic effects which else occurred [21].

A more often investigated kind of substrate is an electrospun nanofiber mat. Such nanofibrous membranes are often used for different biotechnological and medical applications [[22], [23], [24]]. While many biopolymers can be spun from aqueous solutions, the necessary subsequent crosslinking step often requires toxic crosslinkers, resulting in the potential danger to increase cytotoxicity [25]. On the other hand, some polymers such as poly(acrylonitrile) (PAN) can be spun from low-toxic dimethyl sulfoxide (DMSO) [26], making them highly attractive for biotechnological applications. Generally, electrospinning offers the advantages against 3D printing that spinning solutions can be modified easier, e.g. to add nanoparticles or to prepare polymer blends which support cell adhesion and proliferation. Kyrylenko et al. functionalized PAN nanofibers by MXenes and found biocompatibility combined with increased conductivity, which can be supportive for directed cell growth [27]. Wehlage et al. revealed an increase of CHO cell growth on nanospider mats electrospun from PAN blended with gelatin or casein/gelatin, respectively, as compared to pure PAN [28,29]. Lakra et al. showed that gelatin–polyethylenimine (PEI) blend nanofibers with 10 % PEI increased cell adhesion and viability [30]. Other materials from which nanofiber mats for biotechnological applications can be electrospun are, e.g., cellulose, poly(vinyl alcohol) (PVA), chitosan or silk, often doped with nanoparticles to improve wound healing antibacterial properties etc. [[31], [32], [33], [34], [35]].

While the aforementioned 3D printing approaches as well as nanofiber mats mostly consist of polymers, partly with additional ceramics, bone implants are usually made from titanium [36]. Titanium is osteoconductive, i.e. induces formation of new bone material and widely available, making it attractive for the use in orthopedic and dental applications. Nevertheless, the implant surface usually has to be modified to improve osteogenesis, osteoconduction and osteointegration [37].

3D printing, in particular from materials based on titanium has a very large practical use in medicine, due to the possibility of building precise models suited to a given patient (after any imaging method). Currently, a very large part of implants is produced with the use of 3D printing, and the amount is still growing. The combination of 3D printing and electrospinning technology in the aspect of assessing the possibility of growing living tissues will potentially increase the practical areas of using these methods in medicine and the development of this area.

The main aim of our study is the identification of potential substitutes for common well-plates, especially by using 3D printing which allows for producing substrates for cell growth in biotechnology and tissue engineering in various shapes and dimensions. We report the results of a study combining all three aforementioned structures and materials, respectively, for tissue engineering. Titanium substrates with different porosity were 3D printed by laser metal fusion and partly surface-functionalized by a thin electrospun nanofibrous coating. Our results show improved cell adhesion and proliferation of the most porous 3D printed substrate, as compared to commercial Ti sheets as well as common biotechnological well plates.

## 2 Materials and methods

### 2.1 Cell line

The 3T3 Swiss albino mouse cell line was used for cell cultivation. This fibroblast cell line was gained from mouse embryos in 1963 [12] and is often used for stem cell research [38] as well as diverse biotechnological applications, such as reference cells in tests antitumor treatment [39], comparison of primary and immortalized cell lines in drug compatibility tests [40], or wound healing *in vitro* tests [41].

### 2.2 Cell cultivation

For cell cultivation, Dulbecco's Modified Eagle's Medium (DMEM) was prepared by dissolving 12 g/L DMEM/Ham's Nutrient Mixture F12 (DMEM/F12, SAFC Biosciences, Irvine, UK) in 800 ml ultrapure water during stirring for 30 min and adding 2.9 g/L d(+)-glucose (Carl Roth, Karlsruhe, Germany) as well as 0.22 g/L l-glutamine (Applichem, Darmstadt, Germany), followed by adding 2.44 g sodium bicarbonate (NaHCO_3_). Afterwards, the pH value was set to 7.4, before the medium was filled up with ultrapure water to 1 L and stirred for 30 min. After sterile filtration of the medium (Sartolab P, 0.45 μm/0.22 μm, Sartorius, Göttingen, Germany), 100 ml sterile donor horse serum (biowest, Nuaillé, France) were added. The medium was stored at 3 °C.

### 2.3 Substrates

Commercial Ti sheets (Evek GmbH, Mülheim an der Ruhr, Germany), grades 1 and 2, were cut into rectangles of dimensions 2.5 cm × 2 cm. Both grades contain 0.08 % carbon, 0.03 % nitrogen, and 0.015 % hydrogen; grade 1 contains additionally 0.20 % iron and 0.18 % oxygen, while grade 2 has 0.30 % iron and 0.25 % oxygen in addition. Ti grade 1 has a higher corrosion resistance and deformability, while grade 2 has a higher rigidity [42,43]. Both materials are highly biocompatible.

**Fig. 1:**
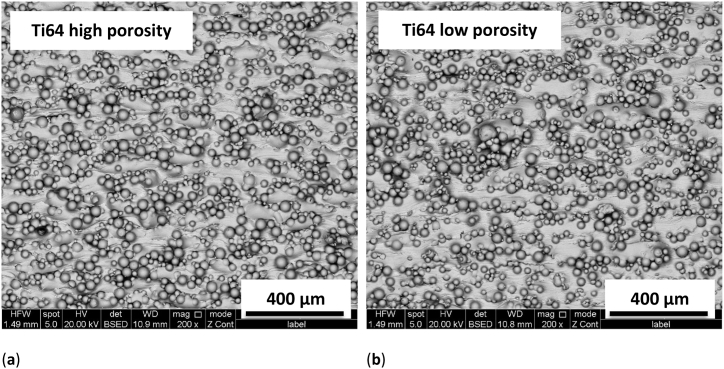
Scanning electron microscopy (SEM) images of 3D printed Ti64 samples: (**a**) prepared with standard parameters for higher porosity; (**b**) prepared with edited parameters for lower porosity. Images were taken by a Quanta 450 FEG microscope (FEI Company, Fremont, CA, USA).

For 3D printing, the Ti alloy Ti6Al4V ELI Gr. 23 (abbreviated as Ti64) was tested which is typically used for medical and aeronautic applications [44] and can, e.g., be prepared by selective laser sintering or electron beam melting [45,46]. It is corrosion resistant and biocompatible and, depending on the printing and post-treatment conditions, slightly hydrophilic or slightly hydrophobic [47]. Here, the Ti64 powder was printed by a TruPrint 1000 laser metal fusion printer (Trumpf, Ditzingen, Germany). The material contains 0.08 % carbon, 0.05 % nitrogen, 0.30 % iron, 0.25 % oxygen, and 0.015 % hydrogen. To produce samples with higher (lower) porosity, the laser speed was set to 905 mm/s (2005 mm/s) and the laser power to 105 W (185 W). This porosity is only related to the 3D printed Ti64 surface, not to a potential additional nanofibrous coating, and was modified since mammalian cells are well-known to adhere differently on surfaces with different roughness and porosity [[48], [49], [50]]. Fig. 1 shows arbitrarily chosen scanning electron microscopy (SEM) images of the surfaces of a sample with the standard parameters to 905 mm/s and 105 W (Fig. 1a) and a sample with edited parameters 2005 mm/s and 185 W (Fig. 1b). On these images, no differences can be recognized. Nevertheless, a difference on smaller scales cannot be excluded.

Some of the titanium substrates were surface-functionalized by electrospinning a thin PAN nanofiber layer in a wirebased electrospinning machine “Nanospider Lab” (Elmarco, Liberec, Czech Republic) on them. In addition to offering a porous interlayer in which mammalian cells can partly penetrate, PAN nanofiber mats are hydrophilic (contact angle ∼ 31°) so that cells can easily attach [51]. The spinning solution contained 16 % PAN (X-PAN, Dralon, Dormagen, Germany) dissolved in DMSO (min. 99.9 %, S3 Chemicals, Bad Oeynhausen, Germany), dissolved by stirring for 2 h at room temperature. Electrospinning was performed with the following spinning parameters: carriage speed 100 mm/s, substrate static, electrode/electrode distance 240 mm, electrode/substrate distance 50 mm, voltage 80 kV, nozzle diameter 0.9 mm, spinning duration 30 s. These solution and spinning parameters are typical for pure PAN and PAN/biopolymer blend nanofiber mats used in previous investigations of mammalian cell growth on electrospun nanofiber mats [28,29]. The voltage is slightly higher than usual in wire-based electrospinning since spinning was only performed for a very short period of time, so that there was not enough time for carefully optimizing the voltage to produce ideal nanofibers, and spinning at this high voltage guaranteed a reproducible layer thickness.

It should be mentioned that PAN nanofiber mats generally adhere well on Ti sheets, as previous tests had shown [52]; however, the adhesion was not investigated in the recent test series where the focus was placed on the question whether an additional nanofibrous coating on Ti substrates would be advantageous for cell adhesion and proliferation.

As a reference, pure nanofiber mats were spun with the aforementioned parameters for 45 min and glued by silicone glue (SILASTIC™ 3D 3335 Liquid Silicone Rubber, from Wacker, Burghausen, Germany) onto cover slips (21 mm × 26 mm) (Carl Roth, Karlsruhe, Germany) to keep them inside the medium during cultivation. The glue was either placed continuously along the whole area or only at the borders of the nanofiber mats.

All substrates were sterilized in an autoclave VX-75 (Systec, Linden, Germany) at 121 °C for 15 min.

The autoclaved specimens were placed in 6-well microtiter plates (Labsolute, Th. Geyer, Renningen, Germany) inside a safety cabinet Safe 2020 (Thermo Electron LED GmbH, Langenselbold, Germany). As references for standard cell adhesion substrates, 3 wells of the 6-well plates were used. 5 ml medium with 192,000 cells were pipetted into each well. Cultivation was carried out during 3 days in a HERAcell 240i incubator (Thermo Electron LED GmbH, Langenselbold, Germany) at 37 °C and 7.5 % CO_2_.

### 2.4 Investigations

Cell adhesion was determined after 3 cultivation days, before the medium was pipetted out of the wells. This relatively short duration was also used in previous investigations and showed clear differences between cell adhesion and proliferation on different electrospun and 3D printed substrates [18,28,29].

Dying was performed using hematoxylin eosin (H&E), applying the H&E Fast Staining Kit (Roth, Karlsruhe, Germany), according to the protocol given by the manufacturer. Firstly, 2 mL H&E solution 1 (modified hematoxylin solution) were pipetted into the wells and remained there for 20 min. The wells were washed 4 times with 4 mL phosphate buffered saline (PBS) buffer, followed by washing with 1 mL 0.1 % hydrochloric acid, and washed 4 times with PBS buffer again. The second part of the dying process was performed by pipetting 2 mL of H&E solution 2 (modified eosin yellow solution) into the wells and left there for 30 s, before the wells were washed for 60 s with 5 mL PBS buffer again, before they were left for drying. All experiments were performed on nine identical specimens in three well-plates per material, with the three free wells serving as a reference.

An inverted microscope Axiovert 40 CFL (Carl Zeiss, Göttingen, Germany) was used to take images of all well grounds and of all substrates, respectively. For each well, micrographs of 10 x 10 tiles were taken at a nominal magnification of 200× in reflected light and afterwards stitched to get 1 image per well.

ImageJ (1.51j8 (National Institutes of Health, Bethesda, MD, USA) was used for image analysis. By converting the images of the dyed cells into grayscale images, optimizing the contrast and the threshold, the area of cell growth was measured in a semi-automated way which is less error-prone than manual measurements on smaller parts of the samples.

The titanium substrates were investigated by AFM measurements using a FlexAFM Axiom (Nanosurf) in tapping mode with Tap190Al g cantilevers. The settings are as follows: setpoint 55 % (for flat substrates) or 70 % (for nanofiber mats), P-gain 550, I-gain 1000, D-gain 100 and vibration amplitude 2 V (for flat substrates) or 6 V (for Ti substrates coated with a nanofiber mat).

Statistical analysis was performed by the Welsh test, valid for independent, normally distributed samples with unequal variances.

## 3 Results and discussion

### 3.1 AFM investigations of sample surfaces

**Fig. 2:**
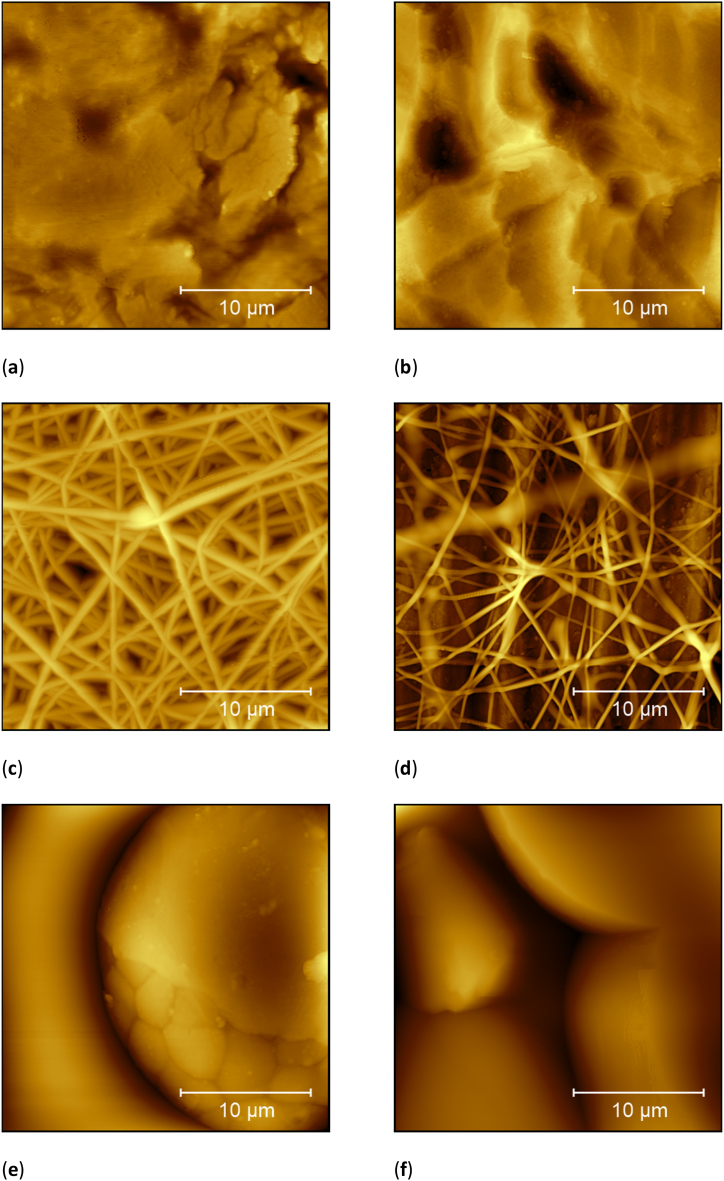
Atomic force microscopy (AFM) images of substrates used in this study: (**a**) Ti grade 1; (**b**) Ti grade 2; (**c**) nanofiber mat on Ti sheet spun for 60 s; (**d**) nanofiber mat on Ti sheet spun for 30 s; (**e**) Ti64 printed with standard parameters; (**f**) Ti64 printed with edited parameters.

Fig. 2 depicts some surfaces of the substrates used in this study, comparing both untreated Ti sheets and a Ti sheet with an additional electrospun nanofiber layer. The 3D printed Ti64 surfaces are too rough for AFM investigation, as could be expected from Fig. 1; they are only added as a comparison (Fig. 2e and f).

Comparing Fig. 2a and b, showing Ti sheets grade 1 and grade 2, respectively, shows clear differences between both surfaces. Ti grade 1 seems to be relatively flat, with several heights of few hundred nanometers diameter. Ti grade 2, on the opposite, is fully structured by grains in the range of some hundred nanometers diameters. The RMS roughness of Ti grade 1 is 437 nm, while the RMS value of Ti grade 2 is 546 nm, both being very similar, as opposed to 2.4 μm for both Ti64 surfaces.

The first tests to modify the surface by a nanofiber coating contained electrospinning for 60 s, the result of which is depicted in Fig. 2c. Here, the cover factor of the nanofibers is quite high, with nearly no holes visible between the fibers. This is why the spinning duration was reduced to 30 s for the next step. The resulting surface is visible in Fig. 2d. Here, separate nanofibers are visible on top of the structured Ti sheet. This structure should enable combining the nanostructured surface – which is well-known to support fibroblast adhesion – with the biocompatible titanium material and was thus used for all nanofibrous coatings used in this study.

### 3.2 Cell growth on Ti sheets

**Fig. 3:**
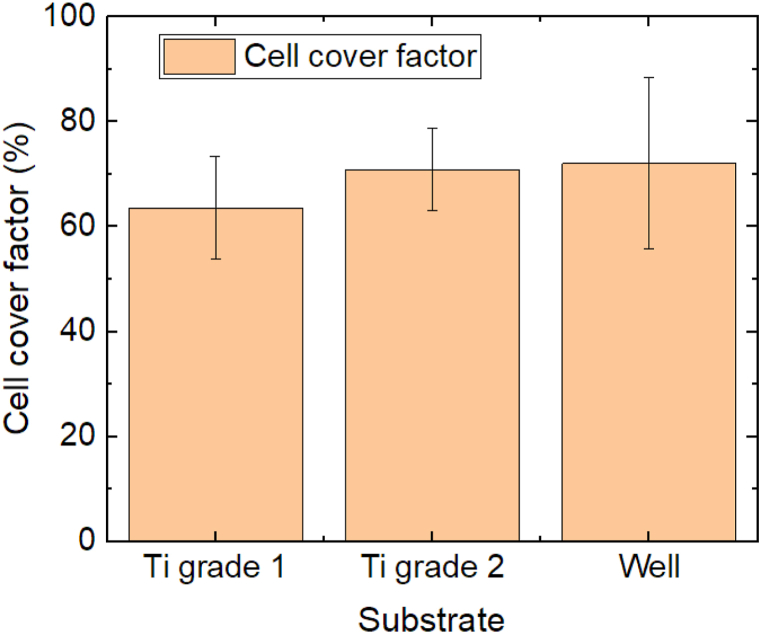
Comparison of the cell cover factors on Ti sheets and on the ground of the plasma-treated polystyrene well. Error bars indicate standard deviations, calculated from 9 specimens per substrate. There are no statistical differences.

Starting with the untreated Ti sheets grade 1 and 2, Fig. 3 shows a comparison of the 3T3 cell growth on these substrates with that on the ground of the well. For the first ones, 3 specimens per substrate were optically investigated along the whole substrate area, while 6 well grounds were examined along their whole area as the reference.

Generally, the rougher Ti grade 2 shows a slightly higher cell cover factor than Ti grade 1, but this difference is not significant. The cell cover factor on the well ground is only marginally larger than on Ti grade 2. This first test series indicates that both titanium sheets show similar proliferation of the 3T3 cells as on the well ground, without any significant differences.

**Fig. 4:**
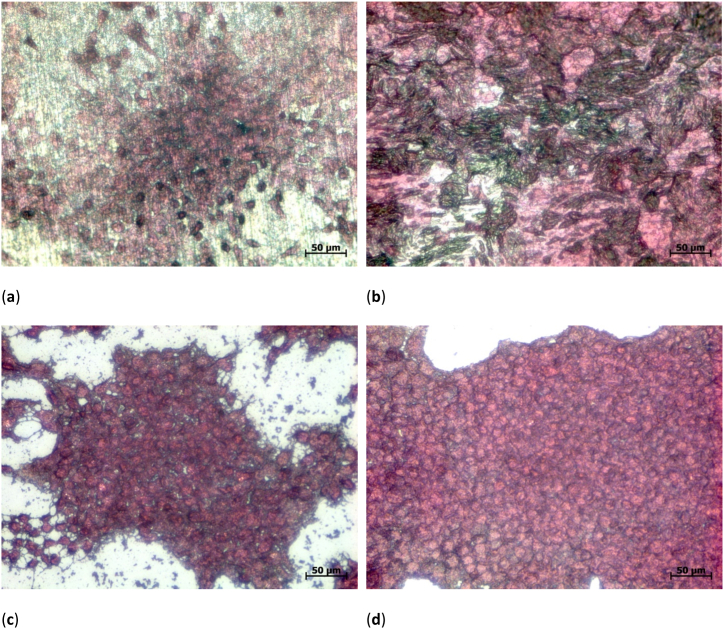
Microscope images of H&E dyed 3T3 cells on different substrates: (**a**) Ti grade 1; (**b**) Ti grade 2; (**c**) well ground (low cell cover factor); (**d**) well ground (high cell cover factor).

Next, Fig. 4 depicts exemplary microscopic images of the dyed 3T3 cells on both titanium sheets as well as on the well ground. On Ti grade 1, the cells have a round shape (Fig. 4a), similar to those visible on the well ground (Fig. 4c and d). Stronger elongated cells are visible on Ti grade 2 (Fig. 4b). This difference can be attributed to the different structures of both Ti sheets and the rougher surface of Ti grade 2. On the more even Ti grade 1, the cells grow more similar to the very even well ground. It should be mentioned that, depending on the position, the well ground can be partially covered, as shown in Fig. 4c, or nearly fully covered with 3T3 cells, as depicted in Fig. 4d. These variations along each well ground, however, are taken into account by measuring the cell cover factor over the whole area. In this way, systematic errors due to “cherry-picking”, i.e. taking images of the best-looking areas [53], is avoided.

### 3.3 Cell growth on Ti sheets with nanofiber coating

**Fig. 5:**
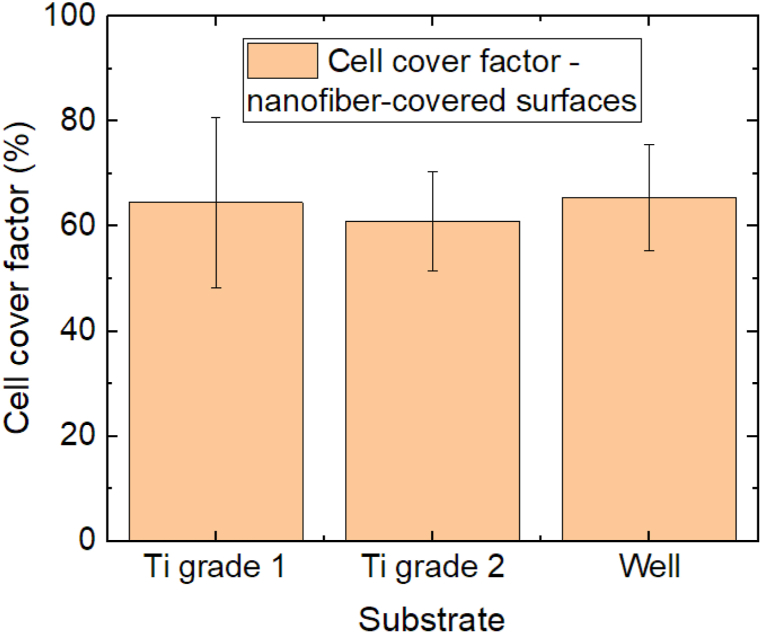
Comparison of the cell cover factors on nanofiber-covered Ti sheets and on the uncovered plasma-treated polystyrene well. Error bars indicate standard deviations, calculated from 9 specimens per substrate. There are no statistical differences.

In the next test series, Ti sheets coated by electrospun nanofibers were investigated in comparison with the well ground, as depicted in Fig. 5. In spite of the nanofibrous surface structure which should support adhesion and proliferation of fibroblasts as the 3T3 cells under investigation, both Ti sheets do not show higher cell cover factors than the well ground used as a reference. Instead, the latter is slightly more covered with cells. These differences are, again, not significant.

**Fig. 6:**
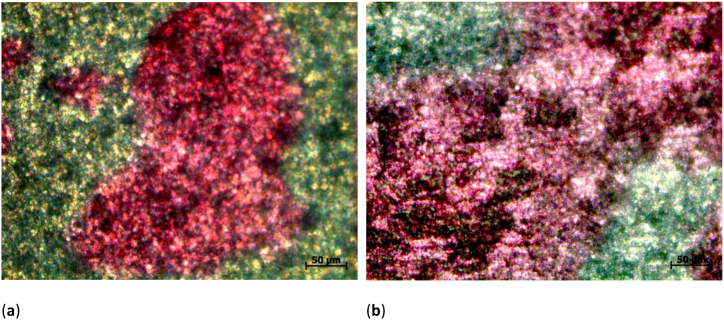
Microscope images of H&E dyed 3T3 cells on different substrates, as statistically evaluated in Fig. 5: (**a**) Ti grade 1 with electrospun nanofibrous coating; (**b**) Ti grade 2 with electrospun nanofibrous coating.

Exemplary images of the samples with dyed 3D3 cells are given in Fig. 6a,b. Generally, images of dyed cells on nanofibrous membranes are hard to take due to the uneven substrate. Besides, the nanostructured surface may even introduce structural colors. Both factors impede evaluating the cell shapes. However, the strong contrast between dyed cells and brighter substrate enable unambiguously measuring the cell cover factor.

### 3.4 Cell growth on nanofiber mats

**Fig. 7:**
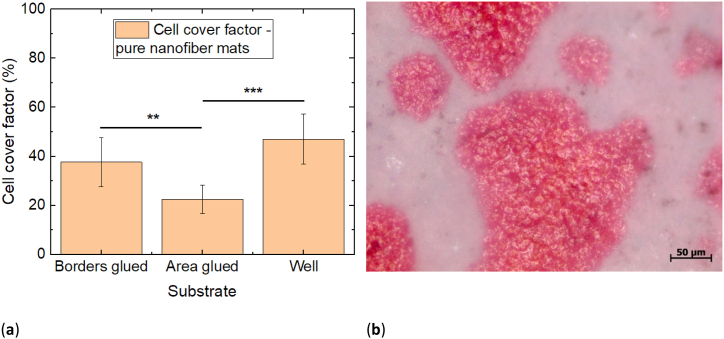
(a) Comparison of the cell cover factors on nanofiber mats glued along the borders of the specimen and along the full area with plasma-treated polystyrene. Error bars indicate standard deviations, calculated from 9 specimens per substrate. Statistical differences are denoted as * (p < 0.05), ** (p < 0.01), or *** (p < 0.001), respectively; (b) microscopic image of H&E dyed 3T3 cells on PAN nanofiber mats glued along the sample borders.

To evaluate adhesion and proliferation of the 3T3 cells on pure PAN nanofiber mats, Fig. 7a shows the cell cover factors on these substrates as compared to the well ground. Firstly, it must be mentioned that the cell cover factor on the well ground is much lower than in the previous tests series. Such differences can always happen when cell cultures are involved, as their growth may be reduced due to diverse environmental factors, which is why each test series contained their own reference for comparison.

Besides, the sample which was fully glued along the whole area of the nanofiber mat shows a significantly reduced cell cover factor, as compared to the cells grown on the well ground. The nanofiber mats which were only glued along the borders show an increased cell cover factor, however, still slightly below the value for the well ground, while this difference is not significant. This finding is in line with previous studies in which pure PAN nanofiber mats showed significantly lower cell proliferation for Chinese hamster ovary (CHO) cells than comparable nanofiber mats from PAN blended with different biopolymers [28,29].

An exemplary microscopic images of the PAN nanofiber mats with attached 3T3 cells can be found in Fig. 7b. Similar to Fig. 6, the nanofibrous membrane surface makes it nearly impossible to differentiate between single cells. In both cases, however, semi-automated differentiation between areas with cells adhered to them and areas where only nanofibers are visible is possible due to the clear color difference.

### 3.5 Cell growth on 3D printed Ti64 substrates

**Fig. 8:**
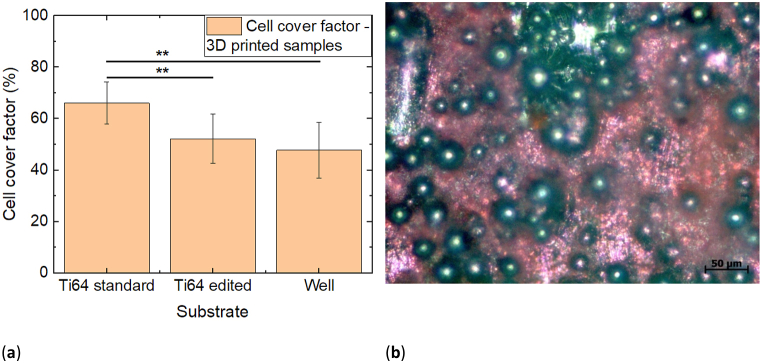
(a) Comparison of the cell cover factors on nanofiber mats glued along the borders of the specimen and along the full area with the ground of the well. Error bars indicate standard deviations, calculated from 9 specimens per substrate. Statistical differences are denoted as * (p < 0.05), ** (p < 0.01), or *** (p < 0.001), respectively; (b) microscopic image of H&E dyed 3T3 cells on 3D printed Ti64 substrates, prepared with standard parameters for higher porosity.

Finally, cell growth on the 3D printed Ti64 substrates was tested. The results are shown in Fig. 8a. Opposite to all other tests before, both kinds of 3D printed substrates show a higher cell cover factor than the well ground. Especially the print with standard parameters for higher porosity allows higher cell attachment and proliferation than the edited Ti64 samples with lower porosity and the well ground, leading to a significant increase in the cell cover factor. Although neither SEM images (Fig. 1) nor AFM roughness calculations (Fig. 2 and text below) showed a difference between both surfaces, there is nevertheless a significant difference visible between the cover factors for 3T3 cells on these samples.

A microscopic image of the cells grown on these substrates is depicted in Fig. 8b. Like in Fig. 1, large beads are visible on both surfaces. The cells are spread over large parts of the surfaces and partly growing in thick enough layers to cover even the top parts of the beads.

Comparing all tests, it can be concluded that the Ti sheets grades 1 and 2, the nanofiber-coated Ti sheets as well as the pure nanofiber mats show lower cell adhesion and proliferation than the well ground which can be regarded as a gold standard. Ti grade 2 shows slightly higher values than Ti grade 1, which can be attributed to its higher biocompatibility and corrosion resistance. Adding a fine nanofiber layer did not increase the cell cover factor. Pure nanofiber mats showed even smaller values, suggesting that investigations of nanofiber mats for cell growth should in the future be performed by directly electrospinning the nanofibrous membrane on a substrate, so that the use of any glue which may impede cell growth can be avoided.

Only the 3D printed Ti64 samples, especially the ones with higher porosity, showed a higher covering by 3T3 cells than the well ground serving as a reference. This result suggests investigating this material further and especially varying the printing parameters in a broad range to optimize the surface porosity for 3T3 and other fibroblast cells. Besides, combining Ti64 with other materials, such as Fe_3_O_4_-graphene oxide or other materials with magnetic, piezoelectric, antibacterial and similar properties may be supportive to improve mammalian cell growth in general as well as especially growth and differentiation of stem cells [[54], [55], [56]].

## 4 Conclusions and outlook

Different substrates were investigated regarding the adhesion and proliferation of 3T3 fibroblastic cells. Commercially available Ti sheets grades 1 and 2 were found to reach lower cell cover factors than the well ground used as a reference. The cell cover factor could not be increased by a thin nanofibrous cover layer. Pure nanofiber mats showed even lower cell adhesion and proliferation, which can most probably be attributed to the silicone glue used to fix them on glass cover sheets. 3D printed Ti64 samples, however, prepared by laser metal fusion, showed significantly improved cell cover factors as compared to the well ground ((48 ± 11)%) under identical conditions, especially for the samples with higher porosity ((66 ± 8)%).

Future investigations should further optimize the 3D printing parameters to increase the surface porosity which might further promote cell attachment and correlate the porosity with cell adhesion and proliferation in order to find the optimum printer settings for the Ti64 material. Next, they should be extended to bone marrow stem cells. Besides, the effect of different sterilization techniques as well as treatments with plasma or UV irradiation should be investigated to further improve adhesion and proliferation of fibroblastic cells on these promising new cell cultivation substrates.

## Funding

This research was partly funded by the German 10.13039/501100006360Federal Ministry for Economic Affairs and Energy as part of the Central Innovation Program for SMEs (ZIM) via the AiF, based on a resolution of the German Bundestag, grant number KK5129703CR0. It was also partly funded by the project Innovative and additive manufacturing technology—new technological solutions for 3D printing of metals and composite materials, reg. no. CZ.02.1.01/0.0/0.0/17_049/0008407, financed by the Structural Funds of the 10.13039/501100000780European Union project. It was co-funded by the European Union under the REFRESH – Research Excellence For REgion Sustainability and High-tech Industries project number CZ.10.03.01/00/22_003/0000048 via the Operational Programme Just Transition. The APC was funded by 10.13039/501100001659Deutsche Forschungsgemeinschaft (10.13039/501100001659DFG, German Research Foundation) – 490988677 – and 10.13039/501100005721Bielefeld University of Applied Sciences and Arts.

## CRediT authorship contribution statement

**Ewin Tanzli:** Writing – review & editing, Visualization, Investigation, Formal analysis, Data curation. **Tomasz Kozior:** Writing – review & editing, Validation, Supervision, Methodology, Formal analysis, Conceptualization. **Jiri Hajnys:** Writing – review & editing, Visualization, Validation, Methodology, Investigation, Formal analysis. **Jakub Mesicek:** Writing – review & editing, Investigation. **Bennet Brockhagen:** Writing – review & editing, Visualization, Methodology. **Timo Grothe:** Writing – review & editing, Methodology. **Andrea Ehrmann:** Writing – original draft, Visualization, Validation, Formal analysis, Conceptualization.

## Declaration of competing interest

The authors declare that they have no known competing financial interests or personal relationships that could have appeared to influence the work reported in this paper.
